# The position of combined medical treatment in acromegaly

**DOI:** 10.20945/2359-3997000000195

**Published:** 2019-11-01

**Authors:** Eva C. Coopmans, Sebastiaan W. F. van Meyel, Aart J van der Lely, Sebastian J. C. M. M. Neggers

**Affiliations:** 1 Section Endocrinology Pituitary Center Rotterdam Erasmus University Medical Center Rotterdam Rotterdam Netherlands Department of Medicine, Section Endocrinology, Pituitary Center Rotterdam, Erasmus University Medical Center Rotterdam, Rotterdam, The Netherlands

**Keywords:** Acromegaly, somatostatin, pegvisomant, pasireotide

## Abstract

Advances in combination medical treatment have offer new perspectives for acromegaly patients with persistent disease activity despite receiving the available medical monotherapies. The outcomes of combination medical treatment may reflect both additive and synergistic effects. This review focuses on combination medical treatment and its current position in acromegaly, based on clinical studies evaluating the efficacy and safety of combined medical treatment(s) and our own experiences with combination therapy. Arch Endocrinol Metab. 2019;63(6):646-52

## INTRODUCTION

Acromegaly is a rare disorder predominantly caused by a growth hormone (GH)-secreting pituitary adenoma, consequently resulting in elevated secretion of insulin-like growth factor-1 (IGF-1) ( 1 ). If untreated, acromegaly leads to disadvantageous metabolic changes and comorbidities, such as hypertension, cardiovascular diseases, diabetes mellitus, respiratory system dysfunction and in particular malignant neoplasms ( 2 - 4 ). There are multiple treatment modalities for acromegaly and the current consensus on the treatment goals include normalization of IGF-1 levels, reduction of GH levels below 1.0 ug/L, decrease of tumour volume, and improvement of clinical signs and symptoms ( 5 , 6 ). Depending on patient characteristics and tumour size the possibilities range from surgery, medical treatment or radiotherapy. Treatment of acromegaly is complex and most cases require a stepwise, multidisciplinary approach to control the disease.

Transsphenoidal surgery (TSS) remains the primary treatment modality in most patients with acromegaly ( 5 , 7 , 8 ), especially in those harbouring a microadenoma or intrasellar macroadenoma ( 9 , 10 ). Surgery is effective in the management of acromegaly and in experienced centers biochemical remission rates up to 80% can be achieved. However, the vast majority of patients have macroadenomas, often with suprasellar extension. In these cases, the postoperative remission rates are much lower ( 11 ). Therefore, in patients for whom surgery is contra-indicated, in whom prefer pharmacological treatment, or in whom postoperative remission is not achieved, additional treatment is needed. This is primarily in the form of medical treatment with radiotherapy generally reserved as a third-line treatment option ( 7 , 12 ).

The medical treatment options are as follows: dopamine agonists (e.g., cabergoline), first-generation long-acting somatostatin receptor ligands (SRLs), the GH receptor antagonist pegvisomant (PEGV) and the second-generation somatostatin multi-receptor ligand pasireotide long-acting release (PAS-LAR). Despite significant medical and surgical advances, many acromegaly patients are not adequately controlled. For these patients advances in combination medical treatment offer new perspectives. The outcomes of combination medical treatment may reflect both additive and synergistic effects. This review aims to discuss the current position of combined medical treatment options in acromegaly. The proposed position of combined medical treatment in acromegaly presented in this review are based on clinical studies evaluating the efficacy and safety of combined medical treatment(s) and our own experiences with combination therapy.

## WHAT IS THE EFFICACY OF COMBINATION THERAPY?

First-line medical treatment according to the recently published consensus criteria of Melmed and colleagues ( 6 ) remains first-generation SRLs monotherapy with lanreotide Autogel (ATG) or octreotide long-acting repeatable (LAR). SRLs act mainly on the somatostatin receptor subtype 2a (SST_2a_) to inhibit GH secretion and normalize both GH and IGF-1 levels with an efficacy rate of about 25%-45% ( 13 - 17 ). If biochemical control is not achieved after administering the maximal dose of first-generation SRLs, the consensus criteria ( 6 ) recommend that treatment should be individualized based on the presence or absence of clinically relevant residual tumour and impaired glucose tolerance. Dopamine agonists monotherapy (e.g., cabergoline), which are acting on dopamine 2 receptors, can be considered as first-line medical therapy postsurgery only for patients with modestly elevated GH and IGF-1 levels (IGF-1 <2.5 x upper limit of normal (ULN) ( 6 , 7 , 18 , 19 ).

There are multiple options for second-line therapy as recommended by the consensus criteria ( 6 ): PAS-LAR monotherapy or PEGV combined with or substituted for first-generation SRLs. PEGV monotherapy has a reported efficacy rate up to 89% ( 20 ). Similarly, PEGV in combination with first-generation SRL has the ability to normalize IGF-1 levels in the majority of patients, provided that the appropriate dose has been applied. According to the consensus criteria, PEGV substituted for or combined with first-generation SRL therapy is recommended for patients with no significant response (<20% IGF-1 reduction) during first-generation SRLs monotherapy ( 6 ). However, we recommend that combination therapy with first-generation SRL and PEGV is the best choice as second-line option in all non-responders (defined as IGF-1 >1.3 x ULN) by using the following arguments ( 21 ):

1. The potential advantage of combination therapy is that a lower PEGV dosage is needed to normalize IGF-1 levels compared with monotherapy of PEGV, leading to a reduction in injection frequency for patients ( 22 - 24 ).
2. There are indications that combination therapy might have a favourable effect on quality of life (QoL) compared to first-generation SRLs monotherapy, including the ones who are biochemically controlled ( 25 ).
3. Although monotherapy with PEGV does not reduce tumour size, combination therapy with first-generation SRL may result in tumour size control or even tumour shrinkage in most patients ( 23 ).
4. Headaches may be alleviated during combination therapy with PEGV and first-generation SRL. It is proposed that nociceptive peptides are inhibited by first-generation SRLs, making it the favourable treatment option for patients with headaches ( 26 , 27 ).

Initiating combination therapy with first-generation SRLs and PEGV is not preferred in patients showing poor control of diabetes during first-generation SRLs monotherapy. In patients receiving first-generation SRLs with worsening of the glucose control, previous studies have shown that PEGV therapy had a more favourable effect on the glycaemic control ( 28 - 30 ). In those cases, PEGV monotherapy would be a more suitable option as it may improve glucose metabolism by reducing insulin resistance ( 28 - 30 ). This is more or less in accordance with the consensus criteria ( 6 ), that recommend patients with pre-existing clinically relevant impaired glucose metabolism should be switched from first-generation SRLs to PEGV monotherapy. In addition, in patients with no biochemical significant response (<20% IGF-1 reduction) and without significant tumour shrinkage (<25% tumour volume reduction) during first-generation SRLs monotherapy, PEGV monotherapy is recommended. The maintenance of first-generation SRLs has potentially no additional benefit and increased the risk of adverse events such as cholelitiasis.

The consensus criteria recommend the addition of cabergoline to continued first-generation SRLs treatment in a small proportion of patients with modestly elevated IGF-1 levels during SRL administration ( 6 ). This recommendation is based on a previous report that showed that IGF-1 normalization has been observed only in patients with modestly elevated IGF-1 levels ( 19 ). The exact mechanism how the combination therapy of cabergoline and first-generation SRL treatment imposes a synergistic effect on the suppression of GH is not known ( 31 - 33 ). Proposed mechanism includes increased intestinal transit time ( 34 ), crosstalk of G-coupled receptors or dimerization on the cell (post-) membrane level ( 35 ) or paradoxical nonobligatory hetero-dimerization ( 36 ). Regarding the efficacy, in a meta-analysis consisting of 77 patients, up to 50% achieved normalized IGF-1 levels after adding cabergoline therapy ( 19 ).

PEGV and cabergoline combination therapy may offer additional benefits to a select group of patients with slightly elevated IGF-1 levels during PEGV monotherapy. However, the use of this combination treatment does not appear to be exceptional in clinical practice, as 10% of acromegaly patients included in the ACROSTUDY ( 37 ) were using this combination therapy. A retrospective study investigated the efficacy of this combination therapy, and found that PEGV in combination with cabergoline normalized IGF-1 levels in 4 out of 14 acromegaly patients (28%) and decreased IGF-1 levels in 9 out of 14 patients (64%) ( 38 ). A significant response to this combination treatment was associated with baseline IGF-1 levels (IGF-1 <1.6 x ULN), female gender, higher baseline prolactin concentrations and a lower body weight ( 38 ). A prospective study investigating PEGV and cabergoline mono- and combination therapy, showed that the combination of low-dose PEGV and cabergoline treatment resulted in 68% of patients in normalization of IGF-1 levels ( 39 ). Moreover, they suggest that this combination treatment is more effective in reducing IGF-1 levels than PEGV or cabergoline treatment alone ( 39 ). Co-administration of PEGV and cabergoline has several advantages over the PEGV and first-generation SRL combination treatment, as cabergoline is orally administered, less expensive than first-generation SRLs and overall well-tolerated.

The current consensus criteria ( 6 ) advocate PAS-LAR monotherapy as second-line treatment for patients without biochemical response to first-generation SRLs if a clinically relevant residual tumour that is unsuitable for resection is present. In accordance with the current consensus criteria ( 6 ), we hypothesize that in particular young patients (aged <40 years) with macroadenomas that show tumour growth during first-generation SRL monotherapy or PEGV [i.e., clinically aggressive tumours ( 40 )], PAS-LAR monotherapy can be considered as a next treatment step before starting with radiotherapy ( 21 ). The same strategy can be applied for patients whose disease was previously not controlled by first-generation SRLs with tumour growth (i.e., reflecting the presence of more aggressive tumours) during PEGV monotherapy ( 21 ). In addition, we recommend to switch to PAS-LAR monotherapy as an alternative to PEGV monotherapy or combination therapy for patients with the following baseline clinical features ( 21 ):

1. Patients whose disease was previously not controlled by first-generation SRLs, who experience side-effects, or who are intolerant to PEGV monotherapy.
2. Patients with severe headaches not responsive to first-generation SRLs. Headaches may be alleviated during PAS-LAR treatment.

There is evidence that biochemical response to somatostatin analogues can be predicted by the SST receptor subtype binding profile in the adenoma tissue ( 41 , 42 ). Whereas first-generation SRLs show a high preferential binding affinity for SST_2a_ receptor and PAS-LAR exhibits particularly high affinity for SST_5_ receptor ( 43 ). However, previous *in vitro* studies ( 44 - 46 ) and one *in vivo* study ( 47 ) suggest that, overall, PAS-LAR exerts its anti-secretory activity mainly by the activation of SST_2a_ receptor. In the latter study, which include patients showing a partial response to first-generation SRLs, the IGF-1 lowering effects of PAS-LAR treatment correlated with the effect of SRL treatment ( 47 ). Therefore, both the clinical response to first-generation SRL treatment and SST_2a_ receptor expression on adenoma tissue could predict the biochemical response to PAS-LAR treatment.

The current consensus criteria ( 6 ) do not address the current position of PAS-LAR in combination with PEGV in the medical management of acromegaly since it was held before our data ( 48 , 49 ) was published. Preferred baseline clinical features for the use of PAS-LAR in combination with PEGV would be patients without diabetes using low PEGV doses (≤80 mg/week) during combination therapy with first-generation SRLs ( 21 ). The PEGV dosages can be reduced or sometimes even discontinued due to the PEGV sparing effect of PAS-LAR ( 48 ). It should be stressed that it is likely that those patients end up with PAS-LAR monotherapy. Patients biochemically controlled during first-generation SRL and PEGV combination therapy, who use first-generation SRLs every three weeks or have symptoms of active acromegaly in the fourth week after first-generation SRL administration, may experience symptomatic improvement after switching to PAS-LAR and PEGV combination therapy ( 21 ). At last, we postulate that PAS-LAR treatment may improve tumour size control or even tumour shrinkage. Therefore, patients experiencing tumour growth during first-generation SRL and PEGV combination therapy could be switched to PAS-LAR and PEGV combination therapy ( 21 ).

The safety of the different combined medical treatments will be addressed in the paragraph below where its effect on tumour size will be discussed separately from other side effects.

## COMBINATION THERAPY AND TUMOUR SIZE

The prevention of further tumour growth and consequently tumour mass complications is an advantageous treatment outcome in acromegaly patients, as the existence of large tumours is associated with poor clinical outcomes ( 50 ). Clinical evidence has shown that first-generation SRL treatment exhibits anti-proliferative effects and induces tumour shrinkage in most patients ( 16 , 51 , 52 ). Moreover, greater tumour shrinkage was observed in naïve patients and those treated with first-generation SRLs monotherapy for more than one year. In addition, dopamine agonists may exert anti-proliferative effects on pituitary tumour cells ( 53 ).

Detailed research on tumour shrinkage and combination therapy is rare. For example, in 158 patients the results of the biochemical efficacy of cabergoline and first-generation SRL combination therapy were available, however, information on tumour volumes is scarce, which could underestimate tumour shrinkage. However, in one study of 10 patients resistant to first-generation SRL therapy ( 54 ), total tumour shrinkage went from 34 to 21 uL (p = 0.009) after the addition of cabergoline (0.25-2 mg/week) for a period of six months. For patients who are biochemically resistant to first-generation SRLs, the addition of cabergoline may be useful as SRLs could maintain long-term effects on tumour mass, while cabergoline may lower IGF-1 levels.

PEGV monotherapy does not have the ability to decrease tumour size. In the Italian ACROSTUDY patients, none of the 35 patients showed significant tumour growth under PEGV alone, whereas in one case, progressive shrinkage of the adenoma was documented by MRI, which was no longer detectable after six years of treatment ( 55 ). In the same study, among the 27 patients treated with PEGV in combination with first-generation SRL, a significant growth of the residual adenoma tissue occurred in one case. However, this patient was characterized by clinically aggressive disease ( 40 ) and, when the tumour enlargement was noted, was eventually treated with PEGV 40 mg/day in combination with lanreotide ATG 120 mg/month ( 55 ). In the Dutch cohort among 141 patients treated with PEGV in combination with first-generation SRL, growth of the adenoma occurred in one case as well, while this growth was already observed before the addition of PEGV ( 23 ). In the report by Neggers and colleagues they assessed the long-term safety of the combined use of PEGV and first-generation SRL in a large group of (n = 86) patients over a longer period of time ( 56 ). In 14 patients the size of the tumour significantly decreased more than 20%, whereas tumour size increase was not observed ( 56 ). All in all, these data do suggest that PEGV does not cause significant increase in tumour size in acromegaly patients. Therefore, PEGV in combination with first-generation SRL should be considered as a safe approach, especially when first-generation SRLs still can reduce tumour size in PEGV treated subjects plus the fact that in a small number of SRL treated subjects, an increase in tumour size has been observed as well.

To our knowledge, there are no studies investigating the tumour response after long-term PAS-LAR alone or in combination with PEGV therapy, except for one. In this follow-up analysis of 47 patients from the PAPE study ( 49 ), which investigated patients who were either treated with PAS-LAR monotherapy or the combination of PAS-LAR and PEGV therapy, assessment of T1 and T2-weighted signal by MRI was performed. Surprisingly, the T2-weighted MRI signal of the adenoma was increased in 14 (30%) of the 47 patients during PAS-LAR treatment ( 57 ). The increase in T2-signal was particularly substantial (>50%) in eight patients, where the majority of the adenoma became T2-hyperintense. Generally speaking, a T2-hyperintense signal indicates cystic degeneration, tumour cell necrosis, or both, which is suggestive of an anti-tumour effect ( 57 ). Besides, they observed clinically significant (≥25%) tumour shrinkage in 5 of the 14 patients with adenomas in which they observed increased T2-signal intensity during 9 months of PAS-LAR treatment. Up to 30 months of PAS-LAR treatment, an additional decrease in tumour volume was observed in the adenomas of five patients (mean tumour volume was 2.9 cm^3^ at baseline, 2.3 cm^3^ after 9 months of PAS-LAR, and 1.9 cm^3^ after about 30 months) ( 58 ).

Antitumour effects of somatostatin receptor ligands have not been reported over the last 30 years. If PAS-LAR could induce cystic degeneration, tumour cell necrosis, or both, it might affect the clinical management of acromegaly. Preoperative treatment with PAS-LAR, for example, might induce (partial) cystic degeneration, tumour cell necrosis, or both, which could improve surgical outcomes. This potential antitumour effect of PAS-LAR might reduce disease activity and even alleviate symptoms, or detrimentally may induce anterior pituitary deficiencies. Therefore, close monitoring of pituitary tumour status is advised when using PAS-LAR therapy alone or in combination with PEGV.

## SAFETY AND QUALITY OF LIFE

First-generation SRLs are generally well tolerated, usually with transient mild side effects. Most patients experience nausea, diarrhoea, gastro-intestinal related complaints and abdominal pain or distension. More severe adverse effects may include hair loss, cholelithiasis and bradycardia ( 13 , 16 ). In general, the net effect of first-generation SRLs on glucose metabolism is considered marginal. Recently, in a large meta-analysis of 47 prospective interventional trials, they studied in 1,297 patients the effect of first-generation SRLs on glucose homeostasis ( 59 ). The authors show that first-generation SRLs significantly reduce insulin secretion, and this was not (completely) counterbalanced by the reduction in IGF-1 and GH levels. Therefore, the net effect of SRLs on glucose metabolism may be clinically relevant, because most patients are already insulin resistant and have prediabetes irrespective of the biochemical control.

Regarding the safety aspects of PEGV monotherapy or in combination with first-generation SRLs, in a recent update of the ACROSTUDY ( 60 ), hepatobiliary-related side effects were found for up to 10% of 2,090 patients, from which 4% are considered related to treatment. From the total of 1,094 patients, 62% remained to have normal transaminase (TA) values during follow-up. There was a reported level of 3% increases in TAs > 3 x ULN. A total of 89 patients entered the study with elevated TAs between 1-3 x ULN, from this 34% drifted downwards to normal TA values during follow-up with PEGV treatment, 46% remained within their baseline range and 10% had TAs > 3 x ULN. Lipodystrophy at the injection site of PEGV has been reported, which regressed in most patients after frequent rotation or after discontinuation of PEGV ( 61 ). In a subgroup of patients, the combination therapy of first-generation SRL and PEGV has favourable effects on QoL compared with first-generation SRL monotherapy, including the patients whose disease is biochemically controlled ( 25 ). Furthermore, an advantage of this combination therapy is that first-generation SRLs are typically effective in resolving headaches due to inhibition of nociceptive peptides, making it the preferred treatment for patients with headaches ( 26 , 27 ).

The adverse effects of the combination therapy of first-generation SRL and cabergoline include: nausea, headache, dizziness and hypotension ( 19 ). High cabergoline dosages used in Parkinson’s patients have valvulopathy as a known side effect ( 62 ). Lower dosages and/or short-term use of dopamine agonists have not been proven to induce valvulopathy in acromegaly patients ( 63 ). Such outcomes of long-term cabergoline and first-generation SRL combination therapy in acromegaly patients is not known.

PAS-LAR monotherapy has a negative effect on glucose metabolism. It suppresses insulin secretion and incretin response, resulting in a higher frequency of hyperglycaemia and diabetes ( 13 , 49 ). In two studies comparing first-generation SRL with PAS-LAR monotherapy, hyperglycaemia-related adverse events were described in 22-30% with first-generation SRLs and in 57%-65% of patients treated with PAS-LAR therapy ( 13 , 14 ). In the PAPE study, the frequency of diabetes mellitus doubled from 33% at baseline to 69% at 24 weeks and increased further to 77% at 48 weeks ( 48 ). The pathophysiology of hyperglycaemia appears to be due to a combination of insulin and incretin suppressive effects of PAS-LAR ( 64 ). However, the precise mechanism of how PAS-LAR causes hyperglycaemia is not well understood. In biochemically controlled patients during first-generation SRL and PEGV combination therapy, who are still experiencing symptoms of active acromegaly in the fourth week after administration of first-generation SRLs, could experience symptomatic improvement after switching to PAS-LAR and PEGV combination therapy.

## CONCLUSION

The desired treatment approach in acromegaly patients should take all the individual traits of the disease into consideration, such as: GH and IGF-1 levels, tumour size, acromegaly symptoms, comorbidities, costs, QoL and patient preferences. Although surgery remains the mainstay treatment, new medical combination therapies have offer new perspectives for acromegaly patients with persistent disease activity despite surgery and/or medical monotherapies. And with the introduction of new effective drugs, the medical therapies may be more suitable for certain types of patients. It is of importance to monitor medical therapies closely as it is crucial in controlling the disease. We elucidated that the use of combination therapies could provide sufficient biochemical or tumour control in patients uncontrolled under first-generation SRL monotherapy. Every medical combination therapy carries along benefits and drawbacks. In the end, the real challenge for the clinician is to decide in each individual patient whether one outweigh other treatment options.
